# Trends in Mental Health Outcomes of College Students Amid the Pandemic (Roadmap mHealth App): Longitudinal Observational Study

**DOI:** 10.2196/67627

**Published:** 2025-01-09

**Authors:** Gautham Jayaraj, Xiao Cao, Adam Horwitz, Michelle Rozwadowski, Skyla Shea, Shira N Hanauer, David A Hanauer, Muneesh Tewari, Kerby Shedden, Sung Won Choi

**Affiliations:** 1 Department of Pediatrics Medical School University of Michigan Ann Arbor, MI United States; 2 Department of Psychiatry Medical School University of Michigan Ann Arbor, MI United States; 3 Department of Learning Health Sciences Medical School University of Michigan Ann Arbor, MI United States; 4 Department of Internal Medicine Medical School University of Michigan Ann Arbor, MI United States; 5 Rogel Comprehensive Cancer Center University of Michigan Ann Arbor, MI United States; 6 VA Ann Arbor Healthcare System Ann Arbor, MI United States; 7 Department of Biomedical Engineering College of Engineering University of Michigan Ann Arbor, MI United States; 8 Center for Computational Medicine and Bioinformatics Medical School University of Michigan Ann Arbor, MI United States; 9 Department of Statistics College of Literature, Sciences, and the Arts University of Michigan Ann Arbor, MI United States

**Keywords:** mHealth, college, student, mental health, positive psychology, flourishing, COVID-19, wellbeing, mobile phone, SARS-CoV-2, coronavirus, pandemic, COVID, app, digital health, smartphone, eHealth, telehealth, telemedicine, longitudinal, higher education, depression, anxiety, loneliness

## Abstract

**Background:**

The mental health crisis among college students intensified amid the COVID-19 pandemic, suggesting an urgent need for innovative solutions to support them. Previous efforts to address mental health concerns have been constrained, often due to the underuse or shortage of services. Mobile health (mHealth) technology holds significant potential for providing resilience-building support and enhancing access to mental health care.

**Objective:**

This study aimed to examine the trends in mental health and well-being outcomes over 3 years among college students, with an exploratory aim to assess the potential impact of the Roadmap mHealth app on these outcomes.

**Methods:**

A fully automated longitudinal observational study was conducted remotely from a large public academic institution in the Midwestern United States, evaluating mental health and well-being outcomes among college students using the Roadmap mHealth app over 3 fall semesters from 2020 to 2022. The study enrolled 2164 college students in Year I, with 1128 and 1033 students returning in Years II and III, respectively. Participants completed various self-reported measures, including the Patient Health Questionnaire-9 for depression, Generalized Anxiety Disorder-7 for anxiety, and additional metrics for coping, flourishing, and loneliness.

**Results:**

The findings indicated an evolving trajectory in students’ mental health. In Year I, depression and anxiety levels were higher compared with levels reported between 2014 and 2019, remaining stable into Year II. However, significant decreases were noted by Year III for both depression (Year I mean 7.78, SD 5.65 vs Year III mean 6.21, SD 4.68; t_108_=–2.90; *P*=.01) and anxiety (Year I mean 6.61, SD 4.91 vs Year III mean 5.62, SD 4.58; t_116_=–2.02; *P*=.046). Problem-focused coping decreased initially from Year I (mean 2.46, SD 0.58) to Year II (mean 2.36, SD 0.60; t_1073_=–5.87; *P*<.001), then increased by Year III (mean 2.40, SD 0.63; t_706_=2.26; *P*=.02). Emotion-focused (Year I mean 2.33, SD 0.41 vs Year III mean 2.22, SD 0.47; t_994_=–7.47; *P*<.001) and avoidant coping (Year I mean 1.76, SD 0.37 vs Year III mean 1.65, SD 0.38; t_997_=–8.53; *P*=.02) consistently decreased. Loneliness significantly decreased from Year I (mean 5.79, SD 1.74) to Year III (mean 5.17, SD 1.78; t_1013_=–10.74; *P*<.001), accompanied by an increase in flourishing from Year I (mean 63.78, SD 14.76) to Year III (mean 66.98, SD 15.06; t_994_=7.22; *P*<.001). Analysis of app usage indicated that the positive piggy bank and gratitude journal were the favored activities. Greater engagement with the app was positively correlated with enhanced flourishing, even after adjusting for demographic and sociobehavioral factors (β=.04, SE .016; t_3974_=2.17; *P*=.03).

**Conclusions:**

In this study, students’ mental health and well-being improved, with notable reductions in depression, anxiety, and loneliness, associated with an increase in flourishing. The app did not appear to worsen students’ mental health. Based on the usage pattern, it is possible the app enhanced positive psychology-based practices. Future research should explore the efficacy of mHealth interventions through randomized controlled trials to further understand their impact on college students’ mental health outcomes.

**Trial Registration:**

ClinicalTrials.gov NCT04766788; https://clinicaltrials.gov/ct2/show/NCT04766788

**International Registered Report Identifier (IRRID):**

RR2-10.2196/29561

## Introduction

Nearly 20 million students are enrolled in colleges across the United States [1]. During this time, students undergo crucial psychological and social development [2]. Mental health issues are prevalent in college student populations, with the peak onset of mental illness occurring before age 25 [3]. However, early detection of emerging mental illness before significant symptoms develop remains limited [4]. Without adequate attention, at-risk young adults with mental health issues are more likely to struggle academically (ie, receive lower grand point averages), drop out of college, or be unemployed compared with their peers [5-7]. These outcomes may consequently correlate with increased substance abuse, self-injury, or suicidality [8-11].

The long-simmering mental health crisis in college students was further exacerbated by the COVID-19 pandemic, which saw higher levels of depression and anxiety than ever previously reported [12,13]. Even before the pandemic, the demand for mental health services was rising [14,15]. Thus, while the current mental health crisis among college students remains a major public health problem, it also presents a unique opportunity to develop and test novel interventions for providing psychosocial support to this population. Psychosocial support refers to interventions aimed at addressing both psychological and social aspects of mental health, helping individuals cope with stress, improve their emotional well-being, and develop supportive social connections [16].

The increase in psychopathology across college campuses has led to expanded counseling and mental health services [17,18]. However, students who stopped attending college due to mental health concerns reported not seeking help or experiencing long wait times [19]. Alarmingly, 80% of students who die by suicide never contact their campus mental health services [20]. Both the underuse and shortage of mental health services are significant issues [21,22]. Mobile health (mHealth) technology offers a novel approach to overcoming barriers associated with mental health care delivery [23]. Evidence suggests that mHealth interventions can support various mental health problems, such as mood disorders, stress, and substance use [24,25]. Focusing on resilience-building aligns with recent recommendations that mental health-related mHealth apps should support well-being, enhance mood, and foster self-care skills rather than address specific mental health problems [26]. Mobile health technology has been shown to be convenient, anonymous, and easy to use [27].

Our interdisciplinary team developed a positive psychology-based mHealth app, Roadmap, initially for use in patients with cancer and their family caregivers. It was initially designed to provide information, education, and skills-based training. Over time, it was iteratively enhanced to provide psychosocial support, that is, the physical, mental, and social health-related quality of life (HRQOL), for any user and to aggregate their step counts and sleep, collected through wearable sensors (Fitbits) [28]. The app incorporated principles based on positive psychology, which emphasizes enhancing individuals’ strengths, cultivating gratitude, fostering resilience, and promoting overall well-being through evidence-based interventions [29,30]. Based on initial observations from our earlier deployment of the Roadmap app, we observed specific patterns in user engagement and feedback regarding the types and frequencies of self-reported measures that were most useful. This informed our strategic selection of validated measures (eg, Patient Health Questionnaire-9 (PHQ-9), Generalized Anxiety Disorder-7 (GAD-7), coping, flourishing, loneliness), as well as the intervals at which these were administered across the 4-month study design. This approach aimed to optimize the app’s utility in monitoring and assessing their health outcomes with app use without excessive burden.

Positive psychology emphasizes enhancing individuals’ strengths, cultivating gratitude, fostering resilience, and promoting overall well-being. Such a framework is particularly suitable for college students, as it aligns with crucial developmental goals during this life stage, such as building resilience, self-efficacy, and effective coping strategies. College students are often navigating significant life changes and stressors, making them an ideal population to benefit from interventions that focus on positive psychology principles. Interventions based on these principles have been developed to improve health (eg, physical, mental, social) and well-being through well-studied constructions (eg, positive daily reflection [29,30], gratitude [31-34], and savoring [35]). Simple strategies aimed at enhancing positive thoughts, emotions, and behaviors have been shown to be effective and highly scalable [36-39]. These strategies have been used in students to support engagement, learning, and well-being. In fact, an economic evaluation of positive psychology strategies that sought to foster positive emotions, stimulate positive functioning, and reduce depressive symptoms found that cost-effectiveness was increased in individuals randomized to the positive psychology intervention arm compared with the waitlisted usual care group [40]. Thus, the strong evidence base for a positive psychology framework to enhance resilience or positive adaptations despite high-risk or chronic stress provides support for their use in college students.

In the fall semester of 2020, we leveraged the Roadmap app to explore HRQOL outcomes among college students [41]. Approximately one-third of students had a mental health disorder and—given the strict lockdown restrictions in the fall semester of 2020—students recorded low physical activity levels (through Fitbits). In addition, we found significant associations of COVID-19 positivity with the use of marijuana and alcohol and with lower belief in public health measures [42]. With consent, the same students were invited to participate in the Roadmap study and report their HRQOL in the following fall semesters of 2021 and 2022. Based on our initial observations [41] and the emerging literature on college student mental health during the pandemic [43], we posited that our participants would report changes in their HRQOL as the pandemic progressed, specifically across the 3-year study period. We also explored whether the Roadmap app, a positive psychology-based mHealth app, might be associated with changes in these HRQOL outcomes. By examining both the trajectory of HRQOL and the role of the Roadmap app, our work contributes to understanding the broader effects of the pandemic on student mental health and highlights the potential of a mHealth app in supporting psychological well-being. The primary aim of this study was to examine the trends in mental health and well-being outcomes over 3 years among college students, with an exploratory aim to assess the potential impact of the Roadmap mHealth app on these outcomes.

## Methods

### Ethical Considerations

**Figure 1:**
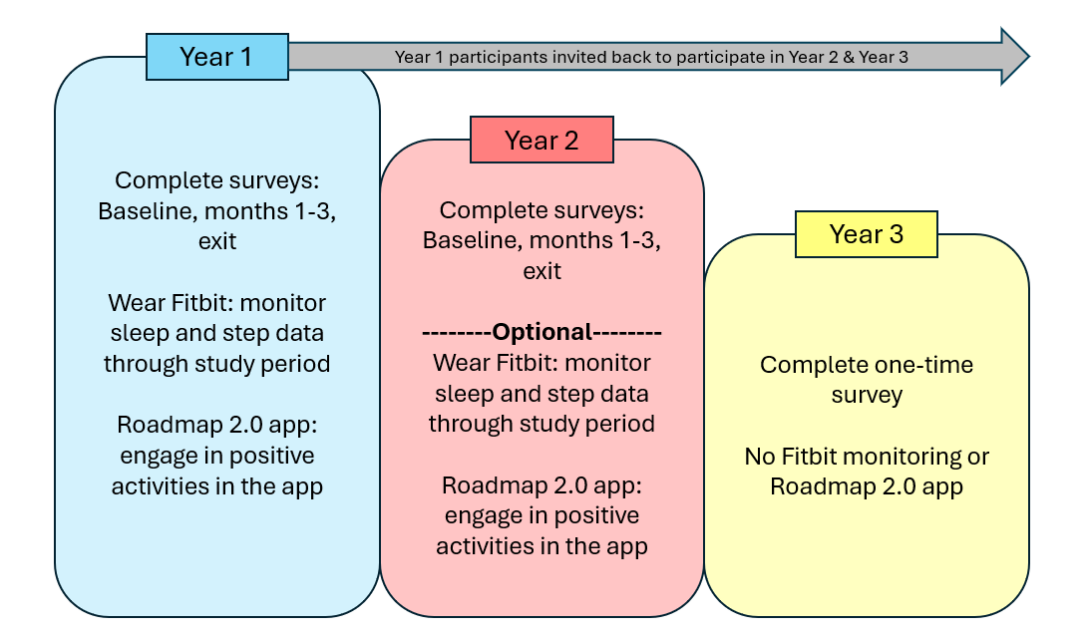
Study schema across all 3 years of the study.

The study was conducted in accordance with the Declaration of Helsinki (as revised in 2013). The study was approved by the institutional review board (IRB) of the University of Michigan Medical School and was registered on ClinicalTrials.gov (NCT04766788). Study participants completed an IRB-approved informed consent through the SignNow platform [44]. The authors are accountable for all aspects of the work in ensuring that questions related to the accuracy or integrity of any part of the work are appropriately investigated and resolved. There were no deviations from the registered study on ClinialTrials.gov NCT04766788. In addition, there were no deviations from the published protocol manuscript [41]. Of note, the study was originally intended to last 1 year (Year I); however, it was expanded to follow the study participants over several years, continuing the following 2 years, designated as Year II and Year III. This was not originally described in the published protocol manuscript [41]. Please refer to the study schema in Figure 1.

### Privacy, Confidentiality, and Compensation

All participant data were anonymized or deidentified to safeguard participant information. Study participants were compensated for their participation. In Year I, participants who completed the baseline and monthly surveys received a US $10 gift card for each survey completed. Each study participant who enrolled received a Fitbit device (valued at US $150) to use during the study and to keep afterward. Year II participants were compensated based on the surveys they completed, which varied in length: US $35 for the baseline survey, US $10 for each monthly survey (up to three surveys), and US $25 for the exit survey. Participants could earn an additional US $10 if they completed all Year II surveys, potentially receiving up to US $100. In addition, those who chose to share their Fitbit data were compensated up to US $5 per week for a maximum of 17 weeks (approximately 4 months), totaling up to US $85. Year III participants received US $25 for completing a one-time survey.

### Study Site

The study took place at a large public academic institution in the Midwestern United States. For the safety of participants and staff during the pandemic, study activities were conducted entirely remotely, with study materials shipped to participants’ residences. This was a 3-phase longitudinal and observational study conducted in the fall semesters of 2020, 2021, and 2022, referred to as Year I, Year II, and Year III, respectively. Notably, the same cohort of students was invited to participate each year, beginning from Year I, aside from any participants who had formally dropped out.

### Recruitment and Enrollment

The research protocol was previously published [41], and additional information regarding the study design for Year I can be found in our previous manuscript [42]. Briefly, eligible participants were required to be college students of at least 18 years of age. Additional eligibility requirements included having access to the necessary resources for participating in a mHealth technology-based intervention (ie, smartphone or tablet and internet access) and being willing to use their personal equipment and internet for the study.

We recruited the Year I cohort of students in the fall semester of 2020 through an email distribution channel managed by the institution and approved by the IRB; this email list initially reached all currently registered and enrolled college students in 2020. In addition, we distributed flyers throughout campus buildings and buses. Interested participants were provided with additional study information, including an overview of study procedures, risks, and benefits. If they met the eligibility criteria, participants were given a digital IRB-approved informed consent form. In Year I, there were 2164 participants enrolled in the study. For Year II and Year III, these students were recontacted in the respective fall semester to complete HRQOL surveys.

### Study Procedures

Roadmap and Fitbit apps: In Year I, participants were instructed to download the Roadmap and Fitbit apps to their smartphones (both free of charge and available on Apple and Google app stores). Each individual participant was provided with a unique Roadmap ID in order to access the app. The Roadmap app offered 8 resilience-based positive activities, previously described in Rozwadowski et al [28] and provided in Multimedia Appendix 1. Participants could also engage with each other anonymously through a Chat Forum (ie, peer-to-peer support) on each of these topics. Participants were instructed to use these activities as much or as little as they found helpful. In addition, participants were asked to record their daily mood on a scale of 1 to 10 (1 being the worst and 10 being the best), with a reminder sent through push notification to their smartphones at 8:00 PM. In Year II, participants had the option to continue using the Roadmap activities to monitor their physical activity and sleep with Fitbits. In Year III, participants did not have access to the Roadmap activities.

### Self-Reported Assessments

Participants self-reported HRQOL assessments through the Roadmap app. The Roadmap app provides a link to Qualtrics (Qualtrics), which hosts study-specific surveys and the secure storage of participant data. We connected participant surveys together using their unique ID associated with the Roadmap app rather than any personally identifiable information. The survey data collected during Year I, Year II, and Year III were used in this study’s analysis. In Year I and Year II, the survey intervals included baseline, Month 1, Month 2, and Month 3; in Year III, the survey was collected only at baseline. At the appropriate intervals, leveraging both Roadmap app reminders and study team emails, we prompted participants to complete each survey and provided compensation for each completed survey. Surveys were scored in accordance with their standardized scoring and as previously published [42] (Textbox 1).

**List of validated self-reported health-related quality of life assessments used in the surveys.**

- Coping: We used the 28-item Brief COPE [45], which assesses the self-reported frequency of use of 14 different coping strategies to deal with a particular situationally specific life event, including self-distraction, active coping, denial, alcohol and drug use, use of emotional support, use of instrumental support, behavioral disengagement, venting, positive framing, planning the use of humor, acceptance, and religion. This study’s analysis followed the subscale analyses provided by Poulus et al 2020 [46].
- Flourishing: A 10-item measure with 2-items each in the following categories, that are (1) happiness and life satisfaction; (2) mental and physical health; (3) meaning and purpose; (4) character and virtue; and (5) close social relationships. A higher score indicates greater flourishing [47].
- Loneliness: A 3-item measure assessing isolation, feeling left out, or lacking companionship. A higher score is associated with increased loneliness [48,49].
- Anxiety: We used the Generalized Anxiety Disorder-7 scale, a 7-item anxiety assessment commonly used in primary care. Scale scores are categorized into minimal, mild, moderate, and severe anxiety. An increased score is associated with increased anxiety. Previous studies found an internal consistency of 0.89 [50].
- Depression: We used the Patient Health Questionnaire-9, a 9-item depression assessment commonly used in primary care. Scale scores are categorized into minimal, mild, moderate, moderately severe, and severe depression. An increased score corresponds to increased depression. Previous studies have reported an internal consistency of 0.83-0.92 [51].

### Statistical Analysis

We analyzed data from the 3 phases, encompassing 2164 students in Year I, with 1128 students returning in Year II and 1033 students returning in Year III. All analyses were performed using the statistical software R (version 4.2.3; R Core Team) and organized in the following manner (Textbox 2).

**List of statistical analyses performed.**

- Descriptive statistics: Descriptive statistics were compiled for categorical variables in terms of frequency and percentage, while continuous variables were summarized using the mean, SD, median, and IQR. Boxplots were used to depict the median and overall distribution of key measures. Univariate associations between continuous and demographic variables were evaluated. All tests were at a significance level of .05.
- Normality assessment and univariate analyses: Depending on data normality (assessed with the Shapiro-Wilk test [52]), continuous variables were compared across 2 categorical variable levels using either 2-sided *t* tests or Wilcoxon rank-sum tests. For comparisons across 3 or more levels of categorical variables, ANOVA or Kruskal-Wallis tests [53] were used. Associations between 2 categorical variables were examined using the chi-square test [54], leveraging the relatively large sample size.
- Correlational analyses: To assess associations between continuous variables, 2-sided Pearson or Spearman correlations [55] were calculated based on data normality.
- Longitudinal analyses: Paired *t* tests were used to evaluate interyear differences in outcome means. Correlation coefficient matrices were constructed to illustrate the relationships among continuous outcomes. We separately considered the monthly responses for flourishing in Year I and Year II. These analyses used a total of 6 assessments, given in consecutive months, modeling the current month’s flourishing for months 2, 3, and 4 on the previous month’s explanatory variables, including app usage. All models included time-invariant fixed effects for time since the pandemic peak (year), sex, undergraduate or graduate status, coping scores, and drug use. To account for within-subject dependence, random intercepts and slopes (year) at the participant level were included.
- Mediation analysis: A directed acyclic graph (DAG) was postulated to relate the key variables of flourishing, depression Patient Health Questionnaire-9 score), anxiety (Generalized Anxiety Disorder-7 score), and loneliness in the context of pandemic time. Based on this DAG, separate regression models were specified for flourishing, anxiety, and depression as outcomes. These models considered only baseline data, modeling the current year’s baseline response in Year II and Year III on the previous year’s baseline explanatory variables. We also conducted a formal mediation analysis based on this DAG.
- Handling missing data: Missing values for continuous variables, including Patient Health Questionnaire-9 and Generalized Anxiety Disorder-7 scores, were imputed using Multiple Imputation by Chained Equation [56] with predictive mean matching.
- Exploratory app use analysis: The app use variable was computed as the sum of the frequencies of positive activities, chat forum interactions, and mood entries.

## Results

### Student Demographics Based on Study Site, Recruitment, and Enrollment

**Figure 2:**
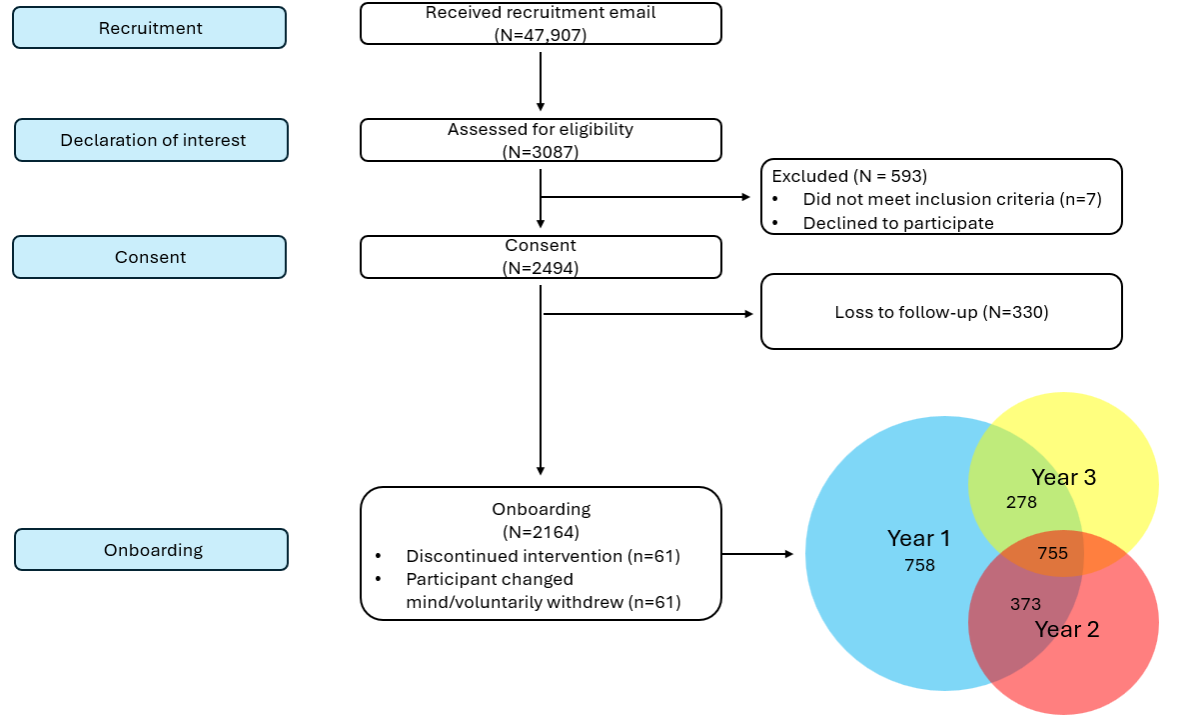
CONSORT (Consolidated Standards of Reporting Trials) flow diagram for participant recruitment and enrollment, accompanied by a Venn diagram illustrating student participation across the 3-year study period. Year I: n=2164; Year II: n=1128; and Year III: n=1033. N=755 students participated in all 3 phases; n=373 were unique to Year I and Year II; and n=278 participants were unique to Year I and Year III.

Of the 2164 students who participated in Year I, 1128 returned in Year II, and 1033 returned in Year III. Notably, 755 students participated in all 3 years, as shown in the CONSORT (Consolidated Standards of Reporting Trials) flow diagram [57] (Figure 2). The majority of participants in Year I were White (1243/2164, 57%), female (1467/2164, 68%), and undergraduates (1429/2164, 66%). The demographic characteristics remained largely consistent across time, with the exception of an increase in those who reported no longer being students and a higher proportion of females (Table 1).

**Table 1:** Demographic breakdown of study participants over 3 years.

Demographics			Participant demographics (population, n %)							*P* value^a^	
			Year I		Year II		Year III				
**School year**											<.001
	Undergraduate	1429 (66.04)		544 (48.23)		338 (32.72)					
	Graduate	719 (33.22)		363 (32.19)		269 (26.04)					
	No longer a student	15 (0.69)		218 (19.32)		426 (41.24)					
	Not available	1 (0.05)		3 (0.27)		0 (0)					
**Sex**											.004
	Female	1467 (67.8)		833 (73.87)		740 (71.64)					
	Male	677 (31.28)		283 (25.09)		283 (27.4)					
	Other	11 (0.51)		11 (0.98)		9 (0.87)					
	Not available	9 (0.42)		1 (0.09)		1 (0.1)					
**Race**											.92
	White	1243 (57.44)		643 (57.03)		604 (58.48)					
	Black or African American	94 (4.34)		40 (3.55)		40 (3.87)					
	Asian	652 (30.13)		356 (31.56)		309 (29.91)					
	Multiracial	113 (5.22)		62 (5.5)		55 (5.32)					
	Other	39 (1.8)		18 (1.6)		18 (1.74)					
	Not available	23 (1.06)		9 (0.8)		7 (0.68)					
**Ethnicity**											.17
	Hispanic or Latino	210 (9.70)		94 (8.33)		81 (7.9)					
	Non-Hispanic or Latino	1949 (90.04)		1029 (91.60)		947 (92.1)					
	Not available	5 (0.23)		5 (0.44)		5 (0.48)					
**Domestic or international**											.36
	Domestic	1996 (92.24)		1027 (91.05)		957 (92.63)					
	International	163 (7.53)		98 (8.7)		74 (7.16)					
	Not available	5 (0.23)		3 (0.27)		2 (0.19)					
**First or continuing generation**											.83
	First generation	541 (25)		290 (25.71)		254 (24.59)					
	Continuing generation	1617 (74.74)		834 (73.94)		775 (75.01)					
	Not available	6 (0.28)		4 (0.35)		4 (0.39)					

^a^*P* values are representative of a chi-square test performed for the entire study population.

### Student Coping Skills Using Descriptive Statistics, Normality Assessment, Univariate Analyses, and Longitudinal Analyses

**Figure 3:**
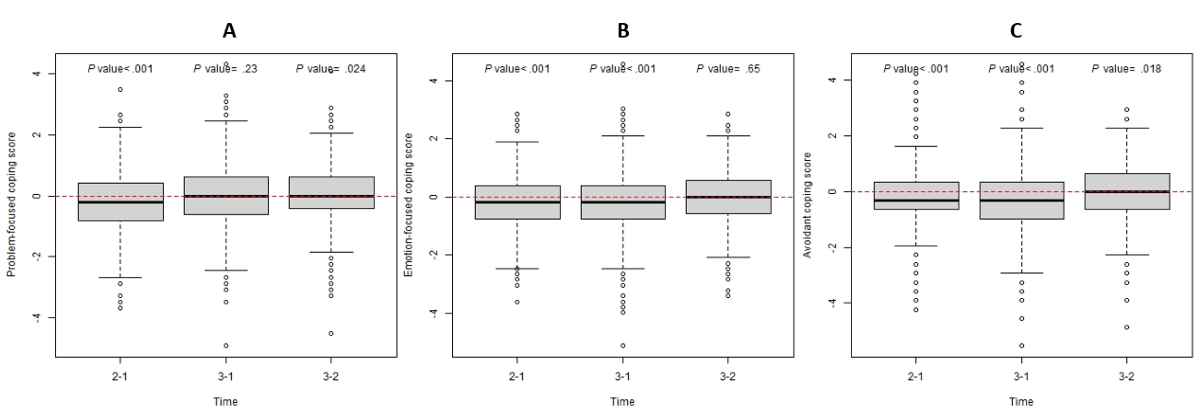
Coping skills across the 3-year study period. The boxes in each plot depict differences between pairs of phases. Panels (A), (B), and (C) illustrate problem-focused, emotion-focused, and avoidant coping, respectively.

We examined student coping skills over the 3-year study period using pairwise comparisons between each pair of years, restricted to students who responded in both years. While problem-focused coping decreased from Year I to Year II (*P*<.001), it remained stable between Year I and Year III (*P=*.23) but increased from Year II to Year III (*P*=.02; Figure 3A). Emotion-focused coping decreased from Year I to Year II (*P*<.001) and from Year I to Year III (*P*<.001) but remained stable between Year II and Year III (*P*=.65; Figure 3B). Avoidant coping decreased across all time points, including from Year I to Year II (*P*<.001), Year I to Year III (*P*<.001), and Year II to Year III (*P*=.02; Figure 3C).

### Student Mental Health and Well-Being Outcomes Using Descriptive Statistics, Normality Assessment, Univariate Analyses, and Longitudinal Analyses

**Figure 4:**
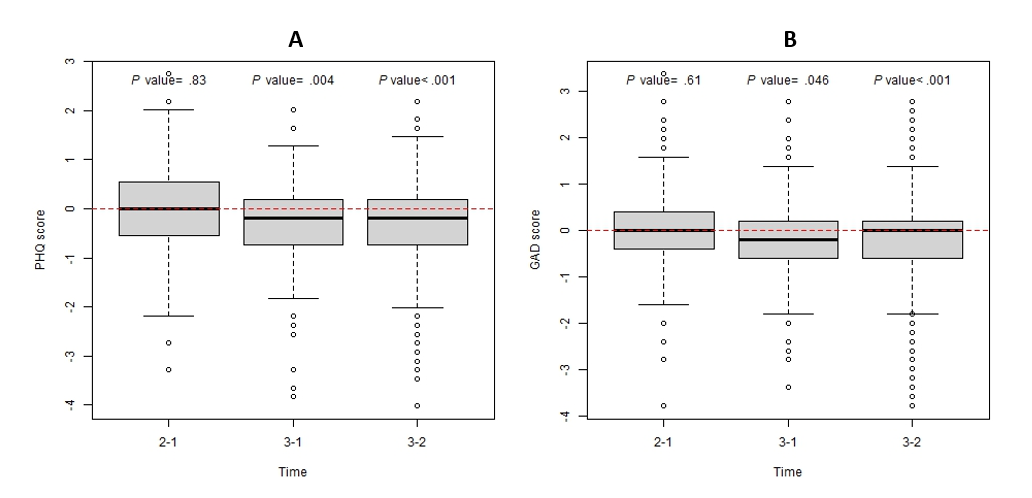
Depression (PHQ-9) and anxiety (GAD-7) across the 3-year study period. GAD-7: Generalized Anxiety Disorder-7; PHQ-9: Patient Health Questionnaire-9.

Next, we examined self-reported outcomes of depression (PHQ-9), anxiety (GAD-7), loneliness, and flourishing. Although there was no change in average depression (Figure 4A) and anxiety symptoms (Figure 4B) between Year I and Year II, both significantly declined from Year I to Year III (*P*=.005 and *P*=.046, respectively) and from Year II to Year III (*P*<.001 and *P*<.001, respectively).

**Figure 5:**
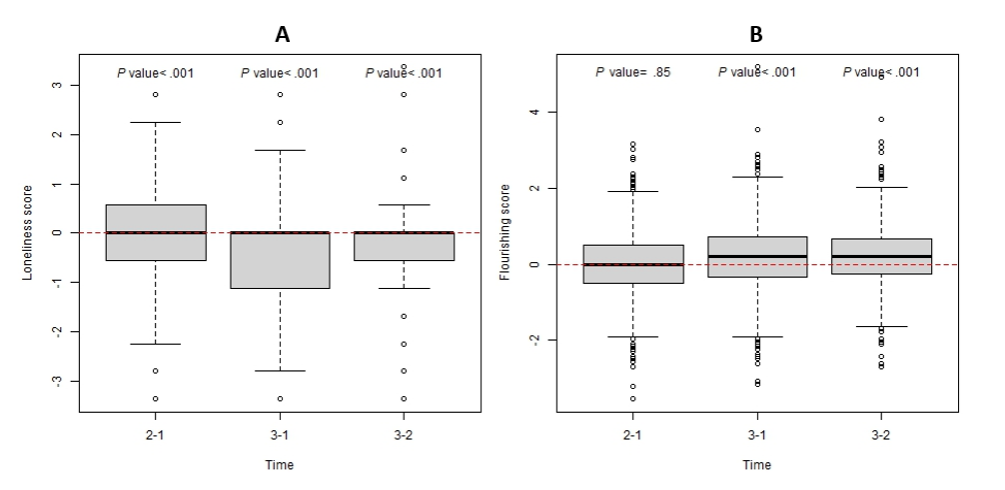
Flourishing and loneliness across the 3-year study period.

The average reported loneliness scores decreased significantly over the 3 study years (*P*<.001 across all pairwise comparisons; Figure 5A). This correlated with increased average flourishing from Year I to Year III (*P*<.001) and from Year II to Year III (*P*<.001; Figure 5B).

### Student Flourishing Assessed by Longitudinal and Mediation Analyses

**Figure 6:**
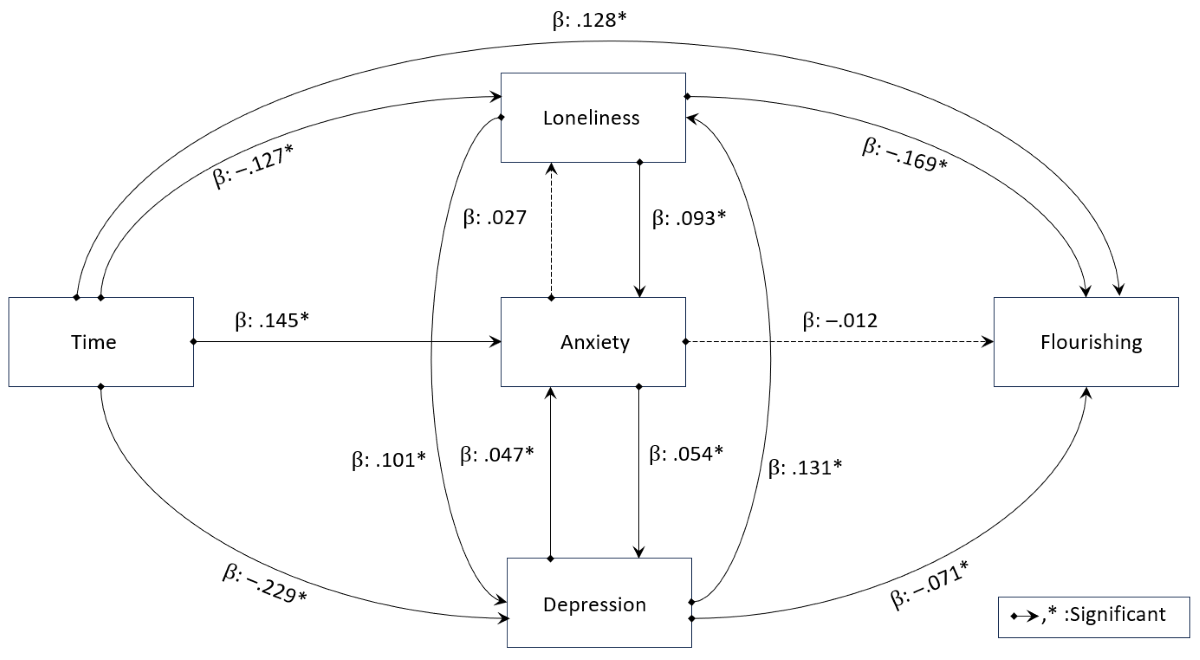
Potential pathways influencing student flourishing. We summarize the multilevel models, where the endpoints of the arrows represent the outcomes, and the starting points indicate the predictors. All predictors in the models are lagged by 1 year, allowing them to forecast the outcomes in the subsequent year. Solid lines with asterisks denote significant *P* values, while dashed lines indicate nonsignificant *P* values. The β symbol represents the estimated value in the respective model.

We explored potential pathways to better understand the factors that may be contributing to changes in students’ flourishing over time using a series of multilevel regression analyses guided by a directed acyclic graph (Figure 6). We found that students’ flourishing improved significantly with time from the peak of the pandemic (β=.13). This improvement was partially influenced by loneliness and depression. That is, in any given year, students with more loneliness or more depression had lower flourishing (β=–.169 and –.071, respectively), while average loneliness, anxiety, and depression also decreased with the passage of time (β=–.127, –.229, and –.145, respectively). Although a formal mediation analysis confirmed the mediating role of loneliness on flourishing, anxiety and depression were not significant mediators of the relationship between pandemic year or severity and flourishing (Multimedia Appendix 2).

### Exploratory App Use Analysis

**Figure 7:**
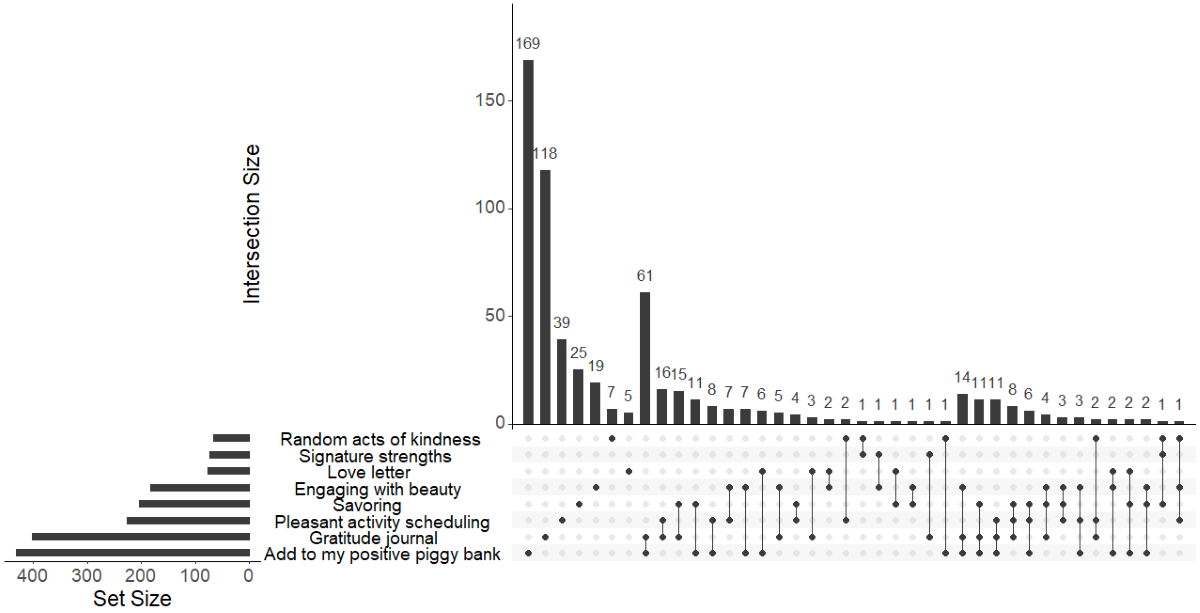
UpSet plot for activity engagement patterns in Year I and Year II.

Next, we examined student app usage patterns over the study period. As shown in the UpSet plot (Figure 7), the 2 most frequently used activities were positive piggy bank and gratitude journal, followed by pleasant activity scheduling, savoring, and engaging with beauty. The least favored activities were love letters, signature strengths, and random acts of kindness.

Single activity usage was the most common (ie, only used that activity and none of the others within a “single” session of using the app), but notable combinations included students who used both positive piggy bank and gratitude journaling. Smaller intersections included combinations, such as (1) pleasant activity scheduling and gratitude journal; (2) engaging with beauty and gratitude journal and positive piggy bank; and (3) savoring gratitude journal and positive piggy bank.

This UpSet plot visualizes the intersection of the positive activities engaged by students using the app in Year I and Year II. The x-axis represents unique combinations of activities, while the y-axis indicates the number of users for each combination. Of note, single app usage is represented by the set size on the left: “Positive piggy bank” (431 unique users) and “Gratitude journal” (402 unique users). Other activities included “Pleasant activity scheduling” (227 users), “Savoring” (205 users), and “Engaging with beauty” (184 users). “Love letter” (78 users) and “Signature strengths” (74 users) had moderate engagement, while “Random acts of kindness” (66 users) were the least used.

Based on the above findings, we were interested in determining whether app use correlated with improved flourishing in the subsequent month. After adjusting for demographic and sociobehavioral characteristics in a longitudinal analysis, more frequent app use was associated with greater flourishing (*P*=.03). For example, implied by Table 2, 30 instances of app use was associated with a nearly 1-unit increase on the flourishing scale. This table presents the estimates and *P* values for the fixed effects in the mixed effects model.

**Table 2:** Fixed effects results from mixed effects model.

Variables			Estimate		*P* value
Problem-focused coping lagged		3.062		<.001	
Emotion-focused coping lagged		–0.870		.08	
Avoidant coping lagged		–1.843		<.001	
PHQ-9^a^ score lagged		–0.213		<.001	
GAD-7^b^ score lagged		–1.151		<.001	
Loneliness score lagged		–1.483		<.001	
Age		.051		.38	
App usage		.035		.03	
**School year**					
	Undergraduate	Reference		Reference	
	Graduate	1.726		<.001	
	No longer a student	1.090		.16	
**Sex**					
	Female	Reference		Reference	
	Male	0.470		.38	
	Other	0.533		.84	
**Ethnicity**					
	No	Reference		Reference	
	Yes	1.262		.18	
**International status**					
	Domestic	Reference		Reference	
	International	2.451		.01	
**Generation**					
	Continuing generation	Reference		Reference	
	First generation	–1.025		.07	
**Tobacco history**					
	No	Reference		Reference	
	Yes	0.086		.91	
**Marijuana history**					
	No	Reference		Reference	
	Yes	–0.646		.18	
**Vaping history**					
	No	Reference		Reference	
	Yes	–0.683		.21	
**Alcohol history**					
	No	Reference		Reference	
	Yes	0.020		.97	
**COVID-19 diagnosis**					
	No	Reference		Reference	
	Yes	–0.115		.93	
**Mental condition**					
	No	Reference		Reference	
	Yes	–4.724		<.001	
**Race**					
	White	Reference		Reference	
	Asian	–3.167		<.001	
	Black	–2.752		.02	
	American Indian and Alaska Native	6.892		.20	
	Multiple	–0.967		.36	
	Other	–1.121		.56	
**Year**					
	2020	Reference		Reference	
	2021	1.941		<.001	
**Month**					
	October	Reference		Reference	
	November	0.820		.005	
	December	0.500		.10	

^a^PHQ-9: Patient Health Questionnaire-9.

^b^GAD-7: Generalized Anxiety Disorder-7.

## Discussion

### Principal Findings

The main finding of this study was the reporting of sustained yet evolving mental health challenges among college students over the 3 years. Depression and anxiety levels of our student population were higher than previously reported levels [12,13,58]; they remained consistent between Year I and Year II (2020-2021) but showed significant improvements by Year III (2022). This trend suggests a potential period of initial adjustments to the pandemic’s disruptions, followed by gradual mental health recovery as students adapted to the new norms and reduced pandemic-related stressors. Before vaccination, students experienced a multitude of changes related to pandemic restrictions, including lockdowns and stay-at-home orders, remote learning mandates, travel bans, social distancing measures, mask mandates, and closure of nonessential businesses [59].

Building on our previous work [41,42], the initial cohort of college students from the 2020 mHealth Roadmap Study were invited to participate in 2 subsequent semesters in the Fall of 2021 and 2022. Leveraging the use of mHealth technology, we longitudinally followed their mental health and well-being outcomes during a historically unprecedented period marked by ongoing global challenges [13]. In this study, we observed changes in student coping strategies over time. Problem-focused coping initially decreased but then increased between Year II and Year III. Emotion-focused coping consistently decreased from Year I, while avoidant coping showed a steady decline across all time points. These adaptive shifts suggest that students may have developed new strategies to manage themselves. It is possible that the type of coping strategy employed may influence mental health outcomes, as previously discussed [60]. A notable and encouraging finding in this study was the significant decrease in reported feelings of loneliness, which was associated with increases in flourishing across the study period. This suggests that students may have reestablished social connections, possibly through remote platforms initially, mHealth technology, and later in-person interactions as restrictions ease.

Students engaged with the Roadmap app with a strong preference for activities focusing on personal reflection, such as a Positive piggy bank and gratitude journal. Our findings suggest that app use may have positively impacted flourishing, which correlated with increased problem-focused coping and reduced avoidant coping. Reduced levels of depression, anxiety, and loneliness may have also played key roles.

### Possible Mechanisms

Counter to our initial hypothesis that mental health outcomes and well-being would worsen over the 3-year study period, our study revealed that, even amid the challenges posed by the pandemic, students reported significant improvements in flourishing over the 3-year period. Flourishing, a term that encapsulates functioning in a manner conducive to growth, resilience, and overall goodness, provides key insights into mental health viewed in a positive manner [61]. This concept aligns with aspects of well-being, including elements such as purpose in life, meaningful relationships, and optimism. Thus, we sought to understand possible mechanisms explaining the link between time from pandemic severity, depression, anxiety, loneliness, and flourishing.

Our data showed that the passage of time from the peak of the pandemic corresponded with higher levels of flourishing. Furthermore, this increased duration was also associated with decreased feelings of loneliness, anxiety, and depression. An increase in flourishing was partially influenced by lower levels of loneliness and depression in the regression model. Interestingly, the mediation analysis only supported the role of loneliness partially mediating increased flourishing, whereas anxiety and depression did not significantly impact the relationship between time since the pandemic’s peak and flourishing. While anxiety had a direct negative effect on depression, anxiety did not influence loneliness. These findings align with ongoing debates about the causal relationships and comorbidity between anxiety and depression [62], as well as their interplay with loneliness [63]. Accordingly, there may be additional factors between loneliness and the constructs of anxiety and depression that were not captured by this study.

Our findings suggest that the time elapsed since the early days of the pandemic may have directly influenced students’ flourishing, possibly due to reasons such as increased rates of vaccination, increased opportunities for social interaction with lifts on social restrictions and mask mandates, and return to in-person classroom settings. It is also possible that app use may have contributed to some of our observations when students had access to the Roadmap’s positive activities in Year I and Year II of the study. During the early period of social restrictions, mHealth technology may have offered an avenue for students to re-establish social connections and practice simple strategies through well-studied constructs, such as positive daily reflection, gratitude, and savoring.

### Comparison with Previous Studies

Previous studies have shown that emotion-focused coping (eg, acceptance, religion, and social support) is associated with improved psychological adjustment, while avoidant coping is linked with increased distress. Problem-focused coping is less used in unpredictable events [64,65] but correlates with positive outcomes [66]. Early in the pandemic, studies indicate that students primarily used emotion-focused coping strategies [65,67,68]. Contrary to these findings, our study participants reported decreased use of problem-focused, emotion-focused, and avoidant coping strategies in the pandemic year (2020). This decline may reflect the widespread use of disengagement strategies at the pandemic’s peak. Over time, students in our study reported a decline in avoidant coping with changes in problem-focused and emotion-focused coping, aligning with other recent pandemic-related studies [65,67,68].

The coping patterns observed herein correlated with an overall decline in depression and anxiety symptoms across each subsequent year of the study. Studies have shown varied findings regarding student psychological distress during the pandemic, with some reporting positive self-efficacy in online learning [69]. However, during the same period (2020-2022), a large national survey of college student mental health (Healthy Minds Network) reported increases in the percentages of students with elevated depression and anxiety scores (PHQ-9: 18%-23%; GAD-7: 31%-37%) [58]. Although we acknowledge differences in participant pools, our study followed the same cohort across the 3 years, while the Healthy Minds Network recruits new students annually; a notable difference is that our students had access to a positive psychology–based mHealth app (Roadmap). In contrast, to our knowledge, access to mHealth app support in the Healthy Minds Network population is not known.

Students who engaged with the Roadmap app favored activities that focused on personal reflection and mindfulness (ie, inward-facing), such as the positive piggy bank and gratitude journal. A recent study showed that mindfulness meditation delivered through mHealth was an approach used by college students to reduce stress and improve mindfulness and self-compassion [70]. Interestingly, we observed lower engagement in activities, such as random acts of kindness or love letters, which involved interaction with others or had an external focus (ie, “outward-facing”). It is possible that during periods of strict lockdown and social distancing, activities requiring physical interaction or outward expressions of kindness, like random acts of kindness or writing love letters, may have been perceived as difficult or impractical. The pandemic created barriers, particularly at colleges, to social interactions, making it harder for students to engage in activities that relied on direct, personal contact or social exchange [71]. During significant life transitions, of both university life as well as the COVID-19 pandemic, it is possible students favored more inward-facing strategies to support their well-being rather than prosocial, outward-facing strategies [72].

### Public Health Impact

The literature suggests that the pandemic may have exacerbated mental health challenges among college students, a population already experiencing increased reports of anxiety, depression, and stress. The experience of social isolation, particularly during a crucial period of social interactions and development growth as emerging adults, may have led to poorer management of other aspects of well-being. Understanding these trends and identifying groups with increased prevalence are crucial for developing novel approaches to mental health care aimed at effectively addressing these issues. To mitigate these challenges, the adoption of novel mHealth technologies through smartphone apps has significantly accelerated over the past several years [73]. Such technologies, as leveraged in this study, hold great potential to fundamentally address mental health challenges, particularly in college students [74].

### Limitations

This study had several limitations. Students were self-selected to be in this study, which was meant to focus on positive psychology-based strategies to enhance mental health. It relied on self-report surveys to collect information about mental health and well-being, and the majority of participants were female. Approximately one-third of the initial participants did not take part in subsequent years of the study (ie, lost to follow-up). In addition, as we followed students over the course of 3 years, some graduated and were no longer on campus, potentially reducing their access to college resources. It is important to note that our findings are specific to students from a single Midwestern location and may not be generalizable to other regions or student populations. For example, certain demographic groups might be underrepresented or overrepresented due to the college’s location in the Midwest. We did not collect data on participants’ ongoing or concurrent mental health treatments or medications. Finally, we acknowledge the lack of a fully powered, randomized controlled trial design. Despite these limitations, this study constitutes a large-scale, longitudinal study conducted during an unprecedented time, providing valuable insights into the mental health challenges of college students.

### Conclusions

This longitudinal study of college students observed a decline in average loneliness, depression, and anxiety with increasing time from the pandemic peak that coincided with increased flourishing. This increased flourishing was partially but not fully explained by improved loneliness, anxiety, and depression. Students had access to a positive psychology-based app and used variable coping strategies during the study. Future research should examine the efficacy of the Roadmap app through a randomized controlled trial, using a robust study design to assess its impact on mental health outcomes. To our knowledge, this study did not produce any unintended consequences through student participation. There were no privacy breaches or technical problems during the study that may have impacted the outcomes.

## Appendix

Multimedia Appendix 1 Roadmap 2.0 app multicomponent features defined.

Multimedia Appendix 2 Mediation analysis result between pandemic year and flourishing.

## Abbreviations

GAD-7: Generalized Anxiety Disorder-7
HRQOL: health-related quality of life
IRB: institutional review board
PHQ-9: Patient Health Questionnaire-9

## Data Availability

All data generated or analyzed during this study are included in this published article and its supplementary information files. Data are available on request. The study protocol and statistical code can be obtained from SWC and XC. The dataset is available from the University of Michigan through a data use agreement.

## References

[1] National Center for Education Statistics.

[2] World Health Organization. Fact Sheets: Adolescent and Young Adult Health.

[3] Coronavirus Disease 2019 (COVID-19). Centers for Disease Control and Prevention.

[4] Colizzi M, Lasalvia A, Ruggeri M (2020). Prevention and early intervention in youth mental health: is it time for a multidisciplinary and trans-diagnostic model for care?. Int J Ment Health Syst.

[5] Hefner J, Eisenberg D (2009). Social support and mental health among college students. Am J Orthopsychiatry.

[6] Arria AM, Caldeira KM, Vincent KB, Winick ER, Baron RA, O'Grady KE (2013). Discontinuous college enrollment: associations with substance use and mental health. Psychiatr Serv.

[7] Lipson SK, Eisenberg D (2018). Mental health and academic attitudes and expectations in university populations: results from the healthy minds study. J Ment Health.

[8] Eisenberg D, Gollust SE, Golberstein E, Hefner JL (2007). Prevalence and correlates of depression, anxiety, and suicidality among university students. Am J Orthopsychiatry.

[9] Gollust SE, Eisenberg D, Golberstein E (2010). Prevalence and correlates of self-injury among university students. Journal of American College Health.

[10] Nock MK, Borges G, Bromet EJ, Cha CB, Kessler RC, Lee S (2008). Suicide and suicidal behavior. Epidemiol Rev.

[11] Mortier P, Auerbach RP, Alonso J, Axinn WG, Cuijpers P, Ebert DD, Green JG, Hwang I, Kessler RC, Liu H, Nock MK, Pinder-Amaker S, Sampson NA, Zaslavsky AM, Abdulmalik J, Aguilar-Gaxiola S, Al-Hamzawi A, Benjet C, Demyttenaere K, Florescu S, De Girolamo G, Gureje O, Haro JM, Hu C, Huang Y, De Jonge P, Karam EG, Kiejna A, Kovess-Masfety V, Lee S, Mcgrath JJ, O'neill S, Nakov V, Pennell BE, Piazza M, Posada-Villa J, Rapsey C, Viana MC, Xavier M, Bruffaerts R (2018). Suicidal thoughts and behaviors among college students and same-aged peers: results from the World Health Organization World Mental health surveys. Soc Psychiatry Psychiatr Epidemiol.

[12] Huckins JF, daSilva AW, Wang W, Hedlund E, Rogers C, Nepal SK, Wu J, Obuchi M, Murphy EI, Meyer ML, Wagner DD, Holtzheimer PE, Campbell AT (2020). Mental health and behavior of college students during the early phases of the COVID-19 pandemic: longitudinal smartphone and ecological momentary assessment study. J Med Internet Res.

[13] Lederer AM, Hoban MT, Lipson SK, Zhou S, Eisenberg D (2021). More than inconvenienced: the unique needs of U.S. college students during the COVID-19 pandemic. Health Educ Behav.

[14] Lipson SK, Lattie E, Eisenberg D (2019). Increased rates of mental health service utilization by U.S. college students: 10-year population-level trends (2007-2017). Psychiatr Serv.

[15] Student Mental Health Innovative Approaches Review Committee Report.

[16] Matsayi Aji L, Baba Muhammad A, Abubakar H, Emel Önal A (2024). Psychosocial Care. Tertiary Care - Medical, Psychosocial, and Environmental Aspects.

[17] Eisenberg D (2019). Countering the troubling increase in mental health symptoms among U.S. college students. J Adolesc Health.

[18] McAleavey AA, Youn SJ, Xiao H, Castonguay LG, Hayes JA, Locke BD (2019). Effectiveness of routine psychotherapy: Method matters. Psychother Res.

[19] Czyz EK, Horwitz AG, Eisenberg D, Kramer A, King CA (2013). Self-reported barriers to professional help seeking among college students at elevated risk for suicide. J Am Coll Health.

[20] Gallagher RP (2006). National survey of counseling center directors 2005. The International Association of Counseling Services (IACS).

[21] Eisenberg D, Downs MF, Golberstein E, Zivin K (2009). Stigma and help seeking for mental health among college students. Med Care Res Rev.

[22] Golberstein E, Eisenberg D, Gollust SE (2008). Perceived stigma and mental health care seeking. Psychiatr Serv.

[23] Bidargaddi N, Schrader G, Klasnja P, Licinio J, Murphy S (2020). Designing m-health interventions for precision mental health support. Transl Psychiatry.

[24] Huckvale K, Nicholas J, Torous J, Larsen ME (2020). Smartphone apps for the treatment of mental health conditions: status and considerations. Curr Opin Psychol.

[25] Lattie EG, Adkins EC, Winquist N, Stiles-Shields C, Wafford QE, Graham AK (2019). Digital mental health interventions for depression, anxiety, and enhancement of psychological well-being among college students: systematic review. J Med Internet Res.

[26] Bakker D, Kazantzis N, Rickwood D, Rickard N (2016). Mental health smartphone apps: review and evidence-based recommendations for future developments. JMIR Ment Health.

[27] Steinhubl SR, Muse ED, Topol EJ (2015). The emerging field of mobile health. Sci Transl Med.

[28] Rozwadowski M, Dittakavi M, Mazzoli A, Hassett AL, Braun T, Barton D, Carlozzi N, Sen S, Tewari M, Hanauer DA, Choi SW (2020). Promoting health and well-being through mobile health technology (Roadmap 2.0) in family caregivers and patients undergoing hematopoietic stem cell transplantation: protocol for the development of a mobile randomized controlled trial. JMIR Res Protoc.

[29] Seligman ME, Steen TA, Park N, Peterson C (2005). Positive psychology progress: empirical validation of interventions. American Psychologist.

[30] Seligman ME, Rashid T, Parks AC (2006). Positive psychotherapy. American Psychologist.

[31] Emmons RA, McCullough ME (2003). Counting blessings versus burdens: an experimental investigation of gratitude and subjective well-being in daily life. J Pers Soc Psychol.

[32] Kashdan TB, Uswatte G, Julian T (2006). Gratitude and hedonic and eudaimonic well-being in Vietnam war veterans. Behav Res Ther.

[33] Moskowitz JT, Hult JR, Duncan LG, Cohn MA, Maurer S, Bussolari C, Acree M (2012). A positive affect intervention for people experiencing health-related stress: development and non-randomized pilot test. J Health Psychol.

[34] Cohn MA, Pietrucha ME, Saslow LR, Hult JR, Moskowitz JT (2014). An online positive affect skills intervention reduces depression in adults with type 2 diabetes. J Posit Psychol.

[35] Bryant FB (2006). A four‐factor model of perceived control: avoiding, coping, obtaining, and savoring. Journal of Personality.

[36] Bolier L, Haverman M, Westerhof G, Riper H, Smit F, Bohlmeijer E (2013). Positive psychology interventions: a meta-analysis of randomized controlled studies. BMC Public Health.

[37] Sin NL, Lyubomirsky S (2009). Enhancing well-being and alleviating depressive symptoms with positive psychology interventions: a practice-friendly meta-analysis. J Clin Psychol.

[38] Hassett AL, Finan PH (2016). The role of resilience in the clinical management of chronic pain. Curr Pain Headache Rep.

[39] Carr A, Cullen K, Keeney C, Canning C, Mooney O, Chinseallaigh E, O’Dowd A (2020). Effectiveness of positive psychology interventions: a systematic review and meta-analysis. The Journal of Positive Psychology.

[40] Bolier L, Majo C, Smit F, Westerhof G, Haverman M, Walburg J, Riper H, Bohlmeijer E (2014). Cost-effectiveness of online positive psychology: randomized controlled trial. The Journal of Positive Psychology.

[41] Cislo C, Clingan C, Gilley K, Rozwadowski M, Gainsburg I, Bradley C, Barabas J, Sandford E, Olesnavich M, Tyler J, Mayer C, DeMoss M, Flora C, Forger DB, Cunningham JL, Tewari M, Choi SW (2021). Monitoring beliefs and physiological measures in students at risk for COVID-19 using wearable sensors and smartphone technology: protocol for a mobile health study. JMIR Res Protoc.

[42] Gilley KN, Baroudi L, Yu M, Gainsburg I, Reddy N, Bradley C, Cislo C, Rozwadowski ML, Clingan CA, DeMoss MS, Churay T, Birditt K, Colabianchi N, Chowdhury M, Forger D, Gagnier J, Zernicke RF, Cunningham JL, Cain SM, Tewari M, Choi SW (2022). Risk factors for COVID-19 in college students identified by physical, mental, and social health reported during the fall 2020 semester: observational study using the Roadmap app and Fitbit wearable sensors. JMIR Ment Health.

[43] Pandya A, Lodha P (2022). Mental health consequences of COVID-19 pandemic among college students and coping approaches adapted by higher education institutions: a scoping review. SSM Ment Health.

[44] Electronic signature that scales with your workflow. signNow.

[45] Carver CS (1997). You want to measure coping but your protocol's too long: consider the brief COPE. Int J Behav Med.

[46] Poulus D, Coulter TJ, Trotter MG, Polman R (2020). Stress and coping in esports and the influence of mental toughness. Front Psychol.

[47] VanderWeele TJ (2017). On the promotion of human flourishing. Proc Natl Acad Sci U S A.

[48] Russell D, Peplau LA, Cutrona CE (1980). The revised UCLA Loneliness Scale: concurrent and discriminant validity evidence. J Pers Soc Psychol.

[49] Hughes ME, Waite LJ, Hawkley LC, Cacioppo JT (2004). A short scale for measuring loneliness in large surveys: results from two population-based studies. Res Aging.

[50] Löwe B, Decker O, Müller S, Brähler E, Schellberg D, Herzog W, Herzberg PY (2008). Validation and standardization of the generalized anxiety disorder screener (GAD-7) in the general population. Med Care.

[51] Cameron Im, Crawford J, Lawton K, Reid I (2008). Psychometric comparison of PHQ-9 and HADS for measuring depression severity in primary care. Br J Gen Pract.

[52] Royston JP (1982). An extension of Shapiro and Wilk's w test for normality to large samples. Applied Statistics.

[53] Bauer DF (1972). Constructing confidence sets using rank statistics. Journal of the American Statistical Association.

[54] Fahrmeir L (2008). Book review: an introduction to categorical data analysis (2nd edition). By A Agresti. Biometrical J.

[55] Best DJ, Roberts DE (1975). Algorithm AS 89: the upper tail probabilities of Spearman's rho. Applied Statistics.

[56] van Buuren S, Groothuis-Oudshoorn CG (2011). mice: multivariate imputation by chained equations in R. J Stat Softw.

[57] Eysenbach G, CONSORT-EHEALTH Group (2011). CONSORT-EHEALTH: improving and standardizing evaluation reports of web-based and mobile health interventions. J Med Internet Res.

[58] Healthy Minds Study among Colleges and Universities. Healthy Minds Network.

[59] Krishnamachari B, Morris A, Zastrow D, Dsida A, Harper B, Santella A (2021). The role of mask mandates, stay at home orders and school closure in curbing the COVID-19 pandemic prior to vaccination. Am J Infect Control.

[60] Horwitz AG, Hill R, King C (2011). Specific coping behaviors in relation to adolescent depression and suicidal ideation. J Adolesc.

[61] Keyes CLM (2002). The mental health continuum: From languishing to flourishing in life. J. Health Soc. Behav.

[62] Cohen JR, Andrews AR, Davis MM, Rudolph KD (2018). Anxiety and depression during childhood and adolescence: testing theoretical models of continuity and discontinuity. J Abnorm Child Psychol.

[63] Hawkley LC, Cacioppo J (2010). Loneliness matters: a theoretical and empirical review of consequences and mechanisms. Ann Behav Med.

[64] Carver C S, Scheier M, Weintraub JK (1989). Assessing coping strategies: a theoretically based approach. J Pers Soc Psychol.

[65] Chu GM, Goger P, Malaktaris A, Lang AJ (2022). The role of threat appraisal and coping style in psychological response to the COVID-19 pandemic among university students. J Affect Disord Rep.

[66] Finkelstein-Fox L, Park C, Riley K (2019). Mindfulness' effects on stress, coping, and mood: a daily diary goodness-of-fit study. Emotion.

[67] Okafor CN, Bautista KJ, Asare M, Opara I (2022). Coping in the time of COVID-19: buffering stressors with coping strategies. J Loss Trauma.

[68] Prasath PR, Mather PC, Bhat CS, James JK (2021). University student well-being during COVID-19: the role of psychological capital and coping strategies. Prof Couns.

[69] Graham MA, Eloff I (2022). Comparing mental health, wellbeing and flourishing in undergraduate students pre- and during the COVID-19 pandemic. Int J Environ Res Public Health.

[70] Huberty J, Green J, Glissmann C, Larkey L, Puzia M, Lee C (2019). Efficacy of the mindfulness meditation mobile app "Calm" to reduce stress among college students: randomized controlled trial. JMIR Mhealth Uhealth.

[71] Sharaievska I, McAnirlin O, Browning M, Larson L, Mullenbach L, Rigolon A, D'Antonio A, Cloutier S, Thomsen J, Metcalf EC, Reigner N (2022). "Messy transitions": students' perspectives on the impacts of the COVID-19 pandemic on higher education. High Educ (Dordr).

[72] Geller L, Greenberg M (2009). Managing the transition process from high school to college and beyond: challenges for individuals, families, and society. Social Work in Mental Health.

[73] Topol EJ (2019). A decade of digital medicine innovation. Sci Transl Med.

[74] Ferrari M, Allan S, Arnold C, Eleftheriadis D, Alvarez-Jimenez M, Gumley A, Gleeson JF (2022). Digital interventions for psychological well-being in university students: systematic review and meta-analysis. J Med Internet Res.

