# Supplementary material for: Trends in Mental Health Outcomes of College Students Amid the Pandemic (Roadmap mHealth App): Longitudinal Observational Study
Source: J Med Internet Res. 2025 Jan 9;27:e67627. doi: 10.2196/67627 (PMC11757984; doi:10.2196/67627)
Supplement: Multimedia Appendix 2 [file jmir_v27i1e67627_app2.docx]

**Multimedia Appendix 2:** Mediation analysis result between pandemic year and flourishing


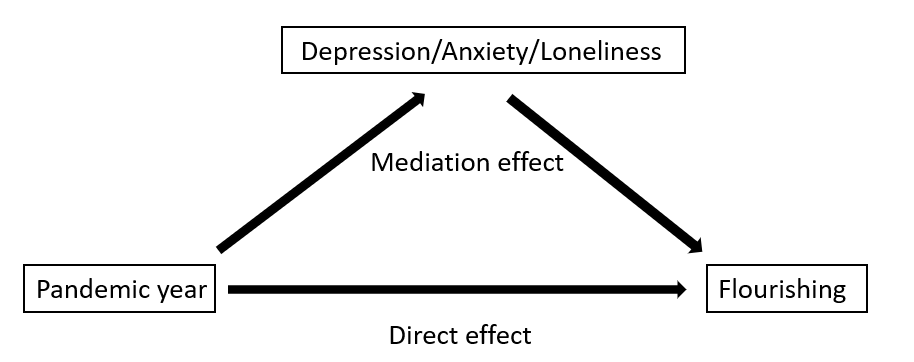


|  | P-value | |  |
| --- | --- | --- | --- |
| Variables | Mediation effect | Direct effect | Proportion of mediation effect |
| Depression (PHQ-9) | 0.36 | <0.001*** | 3.91% |
| Anxiety (GAD-7) | 0.88 | <0.001*** | <0.01% |
| Loneliness | 0.04* | <0.001*** | 9.4% |

Significance codes: 0 ‘***’ 0.001 ‘**’ 0.01 ‘*’ 0.05
